# Mechanical Properties of Re-Entrant Hybrid Honeycomb Structures for Morphing Wings

**DOI:** 10.3390/biomimetics9090521

**Published:** 2024-08-30

**Authors:** Yan Wang, Yingjie Guo, Hui Yang

**Affiliations:** Parallel Robot and Mechatronic System Laboratory of Hebei Province, Yanshan University, Qinhuangdao 066004, China; wangyan_597@163.com (Y.W.); gyj17354287303@163.com (Y.G.)

**Keywords:** biomimetic structures, negative Poisson’s ratio, structural materials, equivalent elastic moduli

## Abstract

The exceptional energy absorption, deformability, and tuneable Poisson’s ratio properties of negative Poisson’s ratio (NPR) honeycomb biomimetic structures make them highly suitable for applications in aerospace, medical, and acoustic stealth industries. The present study proposes a re-entrant hybrid honeycomb (REHH) structure comprising a re-entrant octagonal unit cell and a re-entrant hexagonal unit cell. Theoretical models of the in-plane elastic modulus and Poisson’s ratio are established based on beam theory, and these models are validated through finite element (FE) simulations and tensile experiments conducted on the REHH samples. The influence of the cell geometry parameters on the in-plane elastic behaviours is investigated. The results indicate that the NPR performance of the REHH structure exhibits superior deformation capability compared with the four-point star hybrid honeycomb (FSHH) structure. The experimental REHH structure samples exhibit significant tensile displacement capabilities in the *x*-direction.

## 1. Introduction

Mechanical honeycomb structures refer to a category of artificial composite structures or materials that exhibit exceptional physical properties. By programming the geometrical structure, one can intentionally modify their mechanical properties. Due to the outstanding strength and mechanical characteristics of metamaterials, they exhibit a high capacity for in-plane deformability and out-of-plane bearing capacity. They have numerous engineering applications in aerospace [1,2], medicine [3,4], and acoustic stealth [5,6]. Auxetic honeycomb structures are classified as re-entrant, chiral, and rotating (semi-)rigid structures [7]. Re-entrant structures are extensively utilized in diverse applications due to their excellent energy absorption capabilities and tuneable Poisson’s ratio properties [8]. Olympio [9] developed a zero Poisson’s ratio hybrid and accordion honeycomb structure, whose axial stiffness in the morphing direction did not increase when the skins were restrained in the non-morphing direction. Alsaidi [10] identified the multi-axial stress, strain, and deformation of the skin in a camber morphing aircraft under both structural and aerodynamic loadings, and found an approach that used 3D lattice structures for the skin to be effective.

In the medical field, Hubert Jopek [11] compared the mechanical properties of auxetic structures with origami structures, chiral, and semi-rotary unit cells, and found that the auxetic materials were characterized by the diffusion of Poisson’s ratio and Young’s modulus. Xue [12] addressed the importance of stent structures to clinical outcomes in the treatment of coronary artery disease. An auxetic metamaterial with NPR was introduced into self-expanding stents. Auxetics can enhance the mechanical properties of structures, which helps to resolve the drawbacks due to mechanical failures. Grima-Cornish et al. [13] proposed an auxetic-generating triangular elongation mechanism. This mechanism can generate a giant NPR when it is combined with the well-known rotating squares model. Jopek [14] demonstrated that tubular composite tubes have the advantage of pressure resistance. Auxetics can also be applied to the discipline of acoustic materials. Mustahsan [15] presented a modified re-entrant honeycomb auxetic structure, which was constructed by adding an additional horizontal member between the vertical and re-entrant member of the semi-re-entrant honeycomb model, to obtain higher values of NPR. Structural design methods, acoustic/elastic wave attenuation, and regulation principles were explored by Ma et al. [16]. Metamaterials can be employed as energy-absorbing elements for automotive collisions. Wang et al. [17] proposed a crash box that integrated an outer thin-walled tube with an inner auxetic cellular core; the simulation results showed that the auxetic crash box could improve its energy absorption capacity without increasing the peak impact force too much.

Qi [18] proposed a re-entrant circular configuration by replacing the sloped cell wall of a regular re-entrant honeycomb with circular-arc cell walls, which can dissipate extra energy due to forming more plastic angles during the crushing process. Lu et al. [19] added a narrow rib to the well-known re-entrant cellular structure, which can be considered as a possible basis upon which to design new concepts of auxetic structures with special functions. Fu [20,21] inserted rhombic shapes within the inner re-entrant hexagon, and it exhibited a substantial enhancement in the in-plane Young’s modulus and critical flexural strength compared to the narrow-rib-enhanced unit cells. Xu [22] designed a hybrid unit cell composed of a hexagon and re-entrant hexagon, which had more stable in-plane compression properties. The design methods of hierarchical structures provide new methods for developing novel cellular structures. Tan [23] utilized two novel re-entrant hierarchical sandwich panels, wherein the cell walls of the re-entrant honeycombs were replaced with an isotropic regular hexagon substructure and an equilateral triangle substructure. The two hierarchical cellular structures significantly enhanced the in-plane stiffness and energy absorption capacity. Moreover, the graded sandwich panels effectively mitigated the initial peak force under quasi-static compression. The research on three-dimensional re-entrant structures has gained considerable attention, in addition to that on planar re-entrant structures [24,25]. The fabrication of the complex geometries of 3D re-entrant structures poses challenges when using conventional extrusion and injection moulding processes. The integration of additive manufacturing technology into traditional processes offers a potential solution, enabling the validation of complex structural designs through a theoretical analysis and FE analysis prior to processing [26,27]. In order to enhance the range of variation in Poisson’s ratio for metamaterials, and to improve the out-of-plane stiffness, an investigation is conducted into the mechanical properties of hybrid cellular metamaterials composed of diverse cell types. The challenges for research on hybrid honeycomb structures lie in the following questions. How can we choose an appropriate theoretical framework with which to establish an accurate model for the mechanical properties? How can we select suitable finite element types for simulation and loading, while mitigating boundary effects? How can we ensure high precision during experimental sample processing, where the size of the MIC system marker’s spot diameter determines the minimum sample size, the drawing rate of the drawing system, and how the drawing deformation range corresponds to the simulation?

In this paper, a re-entrant hybrid honeycomb (REHH) structure with a large NPR is proposed, constituted by a re-entrant hexagon (REH) structure and a re-entrant octagon (RE) structure. Theoretical models are developed in Section 2 with which to predict the elastic modulus and Poisson’s ratio of the REHH structures. In Section 3, the accuracy of the theoretical models is validated through FE analysis and experimental tests. Section 4 examines the influence of the various geometrical parameters within the cell on its mechanical characteristics and conducts a comparison of the mechanical properties of the FSHH structures. Section 5 presents the conclusions of the paper.

## 2. Theoretical Analysis Model

**Figure 1:**
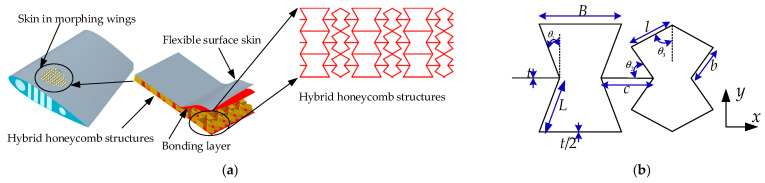
The REHH structures. (**a**) REHH structures in morphing wing, (**b**) geometric parameter.

The proposed REHH structures, as depicted in Figure 1a, adopt a design consisting of REHH structures: an inner REH cell and an inner REO cell. The fundamental support layer of the honeycomb structures is constituted by these two cells. The REHH structure, illustrated in Figure 1b, demonstrate horizontal−vertical symmetry characteristics, with the presence of REH and REO structures. Through periodic arrangement, these cells combine to form the in-plane REHH structures.

The geometric parameters of the REHH structures are depicted in Figure 1b. The design structure is characterized by the following parameters: the length *L* of the inclined wall of the inner REH cell, the length *B* of the horizontal wall of the inner REH cell, the length c of the horizontal wall of the inner REH, as well as the lengths *l* and *b* of the inclined walls of the inner REO cell. Additionally, it includes internal angles *θ*_2_ and *θ*_3_ of the cellular elements, and thickness *t* for the cellular wall. The relative density *ρ_r_* of the REHH strucutres is expressed as follows.

(1) $$\rho_{r}=\frac{\rho^{*}}{\rho_{s}}=\frac{A^{*}}{A_{s}}$$

where ρ* represents the equivalent density of the REHH cell, *ρ_s_* represents the density of the cell material, A* represents the actual bearing area of the cell material perpendicular to the plane of the cell element, A* = *t*(2*c* + 4*L* + 2*B* + 4*b* + 4*l*); *A_s_* is the equivalent section area of the characterization cell, *A_s_* = 2*L_x_L*cos*θ*_1_, which is the two-dimensional projection of the FSHH onto the *xy* plane and covers an area in the *x* and *y* axes; *L_x_* represents the length of the REHH structures in the *x*-direction.

(2) $$\left\{\begin{array}{l} L_{x}=2c+B-2L\sin\theta_{1}+2(l\sin\theta_{3}-b\cos\theta_{2})\\ L_{xd}=\alpha_{2}+0.5\alpha_{1}-\sin\theta_{1}+\alpha_{4}\sin\theta_{3}-\alpha_{3}\cos\theta_{2} \end{array}\right.$$

where *α*_1_ = *B*/*L*, *α*_2_ = *c*/*L*, *α*_3_ = *b*/*L*, *α*_4_ = *l*/*L*, *β* = *t*/*L*, *γ* = *h*/*L* are dimensionless treatments, and *L_xd_* represents a dimensionless reference length, *L_xd_*= *L_x_*/*L*. 

Substituting Equation (2) into Equation (1), the relative density *ρ_r_* of the REHH structures is derived as follows.

(3) $$\rho_{r}=\frac{\beta (\alpha_{2}+2+\alpha_{1}+2\alpha_{3}+2\alpha_{4})}{L_{xd}\cos\theta_{1}}$$

### 2.1. Single-Cell x-Direction Stretching

**Figure 2:**
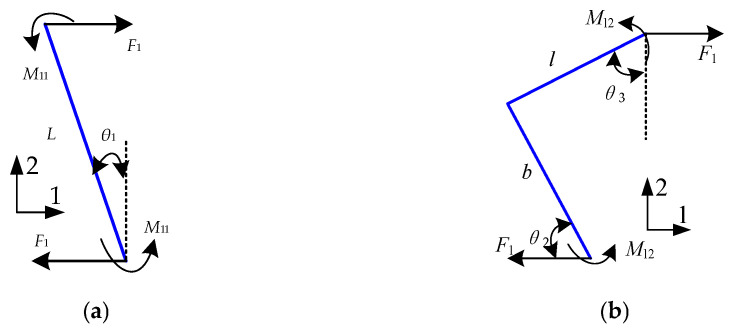
Force on the cell wall along the *x*-axis. (**a**) REH cell, (**b**) REO cell.

The individual cells constituting the REHH structures exhibit symmetry along the *x* and *y* axes, which means that the two cells within the FEHH structures exhibit axisymmetry with respect to two respective coordinates. The selected region for force analysis, as depicted in Figure 2, consisted of a quarter of the cell when subject to a uniform tensile load in the *x*-direction.

The force state of the inner REH cell is depicted in Figure 2a, while the relative equivalent elastic moduli *E*_1*x*_/*E_S_* and Poisson’s ratio *v*_1xy_ of the inner REH cell can be derived from the equations presented in previous papers [8]. The force state of the REO cell is illustrated in Figure 2b, and the theoretical analysis process closely resembles that of the REH structures. The axial force *F*_1_ and bending moment *M*_12_ of the REO can be mathematically expressed as follows:

(4) $$\left\{\begin{array}{l} F_{1}=\sigma_{1}hL\cos\theta_{1}\\ M_{12}=F_{1}(b\sin\theta_{2}+l\cos\theta_{3})/2 \end{array}\right.$$

where *h* is depth of the REHH structures.

The axial force *F_N_*_12_(*x*) and bending moment *M*_12_(*x*) at any given cross-section can be mathematically expressed as follows:

(5) $$F_{N12}(x)=\left\{\begin{array}{l} -F_{1}\cos\theta_{2}(\mathrm{section}\;b)\\ F_{1}\sin\theta_{3}(\mathrm{section}\;l) \end{array}\right.$$

(6) $$M_{12}(x)=\left\{\begin{array}{l} M_{12}-F_{1}x\sin\theta_{2}(\mathrm{section}\;b)\\ M_{12}-F_{1}b\sin\theta_{2}-F_{1}x\cos\theta_{3}(\mathrm{section}\;l) \end{array}\right.$$

The cell is a thin-walled structure, and for the purpose of simplifying the calculations, the shear deformation of the cell can be ignored. The strain energy is derived using the Euler–Bernoulli beam model and Cartesian Second Theorem.

(7) $$U=U_{M}+U_{N}=\int_{L}(M^{2}/2E_{S}I+{F_{N}}^{2}/2E_{S}A)dx$$

where *M* represents the internal bending moment, *F_N_* represents the axial force, *E_S_* denotes the Young’s modulus of the honeycomb material, *A* represents the cross-sectional area of the REHH cellular wall, *A* = *ht*, and *I* represents the moment of inertia of the REHH cellular wall, *I* = *ht*^3^/12.

The strain energy of the inner REO quarter-cell cell in the deformed state can be determined by substituting Equations (5) and (6) into Equation (7):

(8) $$U_{12}=\frac{{F_{1}}^{2}}{24E_{S}I}U_{12M}+\frac{{F_{1}}^{2}}{24E_{S}A}U_{12N}$$

where *u*_12*M*_ = *b*^3^sin^2^*θ*_2_ + 3*bl*^2^cos^2^*θ*_3_ + *l*^3^cos^2^*θ*_3_ + 3*b*^2^*l*sin^2^*θ*_2_, *u*_12*N*_ = *b* cos^2^*θ*_2_ + *l*sin^2^*θ*_3_.

The total deformation *δ*_2*x*_ and the total strain *ε*_2*x*_ are obtained by calculating partial derivatives in accordance with Carpenter’s second theorem.

(9) $$\delta_{2x}=\frac{2F_{1}}{12E_{S}I}U_{12M}+\frac{2F_{1}}{E_{S}A}U_{12N}$$

(10) $$\varepsilon_{2x}=\frac{\delta_{2x}}{2l\sin\theta_{3}-2b\cos\theta_{2}}$$

The equivalent elastic moduli *E*_2*x*_/*E_S_* of the inner REO structures can be calculated by employing Equations (5), (11) and (12).

(11) $$E_{2x}=\frac{\sigma_{1}}{\varepsilon_{2x}}$$

(12) $$\frac{E_{2x}}{E_{S}}=\frac{l\sin\theta_{3}-b\cos\theta_{2}}{hL\cos\theta_{1}(\frac{U_{12M}}{12I}+\frac{U_{12N}}{A})}$$

**Figure 3:**
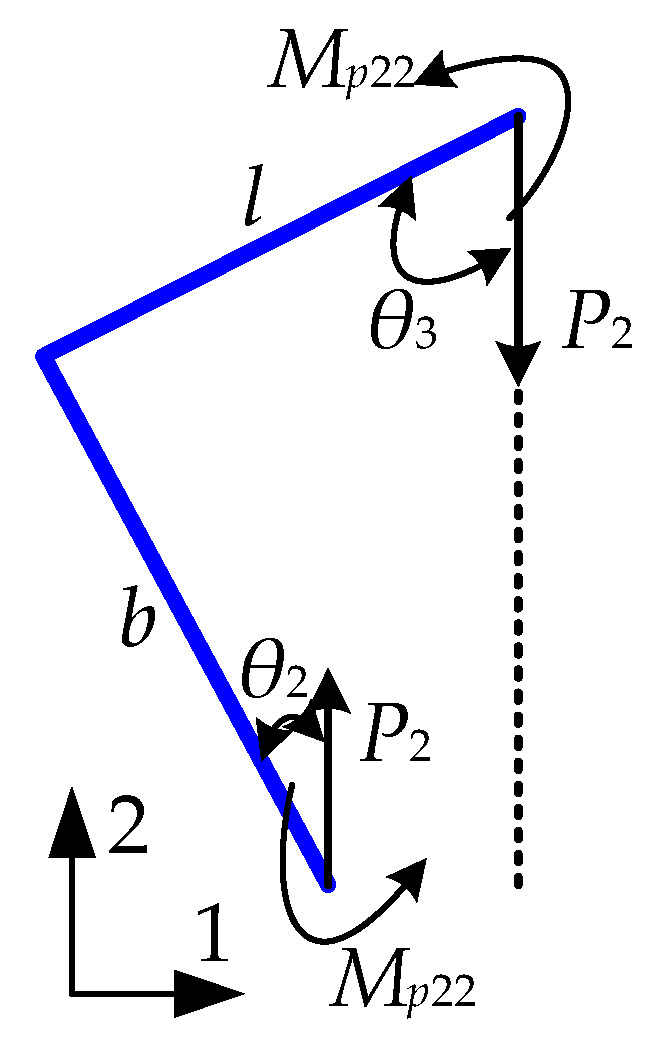
REO structure unit load force.

The force analysis of the REO quarter-cell wall, utilizing the unit load method, is depicted in Figure 3.

The displacement *δ*_2*x*−*y*_ is generated by the unit virtual force *P*_2_ and bending moment *M*_P22_, thereby allowing for determination of the strain *ε*_2*x*−*y*_ based on the deformation geometry:

(13) $$\delta_{2x-y}=\frac{u_{b}}{EA}+\frac{u_{1}}{6EI}$$

where *u_b_* = *F*_1_*b*sin*θ*_2_cos*θ*_2_−F_1_*l*sin*θ*_3_cos*θ*_3_, *u*_1_ = 3*M*_12_*b*^2^cos*θ*_2_ − 2*F*_1_*b*^3^sin*θ*_2_cos*θ*_3_ + 3*M*_12_*l*^2^sin*θ*_3_3*F*_1_*l*^2^ sin*θ*_2_sin*θ*_3_ −2F_1_*l*^3^sin*θ*_3_cos*θ*_3_.

(14) $$\varepsilon_{2x-y}=\frac{-\delta_{2x-y}}{l\cos\theta_{3}+b\sin\theta_{2}}$$

The Poisson ratio can be determined by employing Equations (11) and (15).

(15) $$\nu_{2xy}=-\varepsilon_{2x-y}/\varepsilon_{2x}=2\delta_{2x-y}/\delta_{2x}$$

### 2.2. Single-Cell y-Direction Stretching

**Figure 4:**
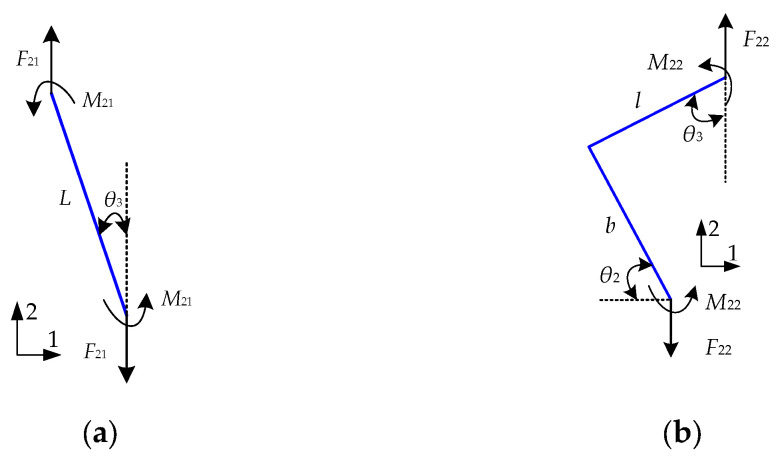
Force on the cell wall along the *x*-axis. (**a**) REH quarter cell, (**b**) REO quarter cell.

When a uniform tensile load is applied in the *y*-direction to the REHH structures, Figure 4 illustrates the forces exerted on the quarter cell. The relative equivalent elastic moduli *E*_1*y*_/*E_S_* and Poisson’s ratio *v*_1*yx*_ can also be calculated using methods described in reference [8]. The force state of the REO cell is shown in Figure 4b, following the theoretical analysis process parallels with that of its REH cell. The axial force *F*_22_ and bending moment *M*_22_ of the REO cell can be expressed as follows.

(16) $$\left\{\begin{array}{l} F_{22}=\delta_{2}h(l\sin\theta_{3}-b\cos\theta_{2})\\ M_{22}=F_{22}.(l\sin\theta_{3}-b\cos\theta_{2})/2os\theta_{3})/2 \end{array}\right.$$

The axial force *F_N_*_22_(*x*) and bending moment *M*_22_(*x*) at an arbitrary cross-section can be derived as follows:

(17) $$\left\{\begin{array}{l} F_{N22}(x)=F_{22}\sin\theta_{2}(\mathrm{for}\;\mathrm{segment}\;b)\\ F_{N22}(x)=F_{22}\cos\theta_{3}(\mathrm{for}\;\mathrm{segment}\;l) \end{array}\right.$$

(18) $$\left\{\begin{array}{l} M_{22}(x)=M_{22}-F_{22}x\cos\theta_{2}(\mathrm{for}\;\mathrm{segment}\;b)\\ M_{22}(x)=M_{22}-F_{22}b\cos\theta_{2}-F_{22}x\sin\theta_{3}(\mathrm{for}\;\mathrm{segment}\;l) \end{array}\right.$$

The *y*-direction deformation *δ*_2*y*_ and strain *ε*_2*y*_ of the inner REO cell under the unit load method are determined in a similar manner as the *x*-direction analysis.

(19) $$\delta_{2y}=\frac{2F_{22}}{12E_{s}I}u_{22M}+\frac{2F_{22}}{E_{s}A}u_{22N}$$

(20) $$\varepsilon_{2y}=\frac{\delta_{2y}}{2b\sin\theta_{2}+2l\cos\theta_{3}}$$

where *u*_22*M*_ = *b*^3^cos^2^*θ*_2_ + (3*bl*^2^ + *l*^3^) × sin^2^*θ*_3_ + 3*b*^2^*l*cos^2^*θ*_2_, *u*_22N_ = *b*sin^2^*θ*_2_ + *l*cos^2^*θ*_3_.

The relative equivalent elastic moduli *E*_2*y*_/*E_S_* for the inner REO cell can be obtained by combining Equations (17) and (21).

(21) $$\frac{E_{2y}}{E_{S}}=\frac{\delta_{2}}{\varepsilon_{2y}E_{S}}=\frac{b\sin\theta_{2}+l\cos\theta_{3}}{h(l\sin\theta_{3}-b\cos\theta_{2})\cdot (\frac{U_{22M}}{12I}+\frac{U_{22N}}{A})}$$

(22) $$\varepsilon_{2y}=\frac{\delta_{2y}}{2b\sin\theta_{2}+2l\cos\theta_{3}}$$

where *u*_12_ = 1/2 × *M*_22_(*b*^2^sin*θ*_2_ + *l*^2^cos*θ*_3_) − F_22_(1/6 × *b*^3^sin*θ*_2_ + *bl*^2^cos*θ*_2_cos*θ*_3_ + 1/6 × *l*^3^sin^2^*θ*_3_), *u_b_*_2_ = *F*_22_ (*b*sin^2^*θ*_2_ − *l*sin^2^*θ*_3_).

(23) $$\varepsilon_{2y-x}=\frac{-\delta_{2y-x}}{\left(l\sin\theta_{3}-b\cos\theta_{2}\right)}$$

The Poisson’s ratio *v*_2*yx*_ for the inner REO structures is determined by combining Equations (21) and (24), which is written as follows:

(24) $$v_{2yx}=-\varepsilon_{2y-x}/\varepsilon_{2y}$$

### 2.3. Hybrid Honeycomb Structure Elastic Modulus and Poisson’s Ratio

**Figure 5:**
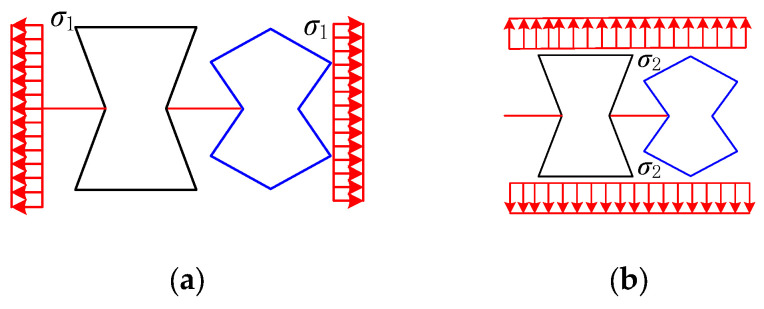
Equivalent stiffness of hybrid cell elements. (**a**) Loading in *x*-direction, (**b**) loading in *y*-direction.

The REHH cell can be considered as a combination of series and parallel springs, as represented in Figure 5, when subjected to the uniform load of *σ*_1_ and *σ*_2_ in the *x* and *y* directions. The total stiffness of the structure can be mathematically represented as follows:

(25) $$\left\{\begin{array}{l} K_{x}=\frac{K_{1x}K_{2x}}{K_{1x}+K_{2x}}\\ K_{y}=K_{1y}+K_{2y} \end{array}\right.$$

**Figure 6:**
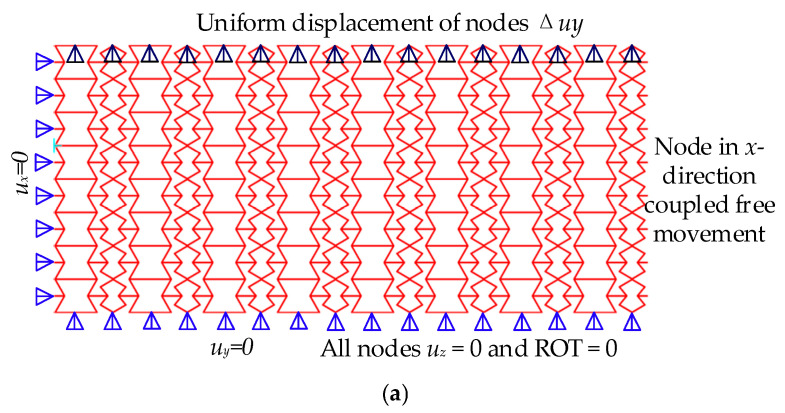

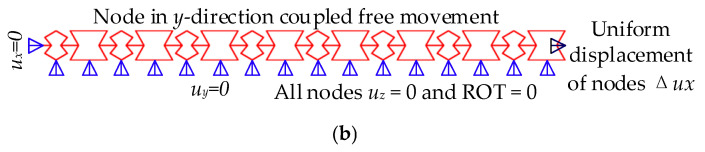
Boundary condition setting. (**a**) Loading boundary conditions in *y*-direction, (**b**) loading boundary conditions in *x*-direction.

The structures can be considered as a series of springs when subjected to loading in the *x*-direction, as illustrated in Figure 6. The spring stiffness values, *K*_1*x*_ and *K*_2*x*_, along with the overall equivalent stiffness *K_x_*, can be determined for the REH cell and the REO cell in the *x*-direction using Hooke’s law.

(26) $$\left\{\begin{array}{l} K_{1x}=\frac{F_{x}}{\delta_{1x}}=\frac{E_{1x}\gamma L\cos\theta_{1}}{\left(\alpha_{2}+\alpha_{1}/2-\sin\theta_{1}\right)}\\ K_{2x}=E_{2x}\gamma L\\ K_{x}=\frac{E_{x}\gamma L\cos\theta_{1}}{L_{xd}} \end{array}\right.$$

The relative equivalent elastic moduli *E*_1*x*_/*E_s_* in the *x*-direction and *y*-direction of the inner REH structures, as derived in the literature of *J_s_*, *E*_1*y*_/*E_s_*, are presented as follows:

(27) $$\left\{\begin{array}{l} \frac{E_{1x}}{E_{S}}=\frac{\beta^{3}(\alpha_{2}+\alpha_{1}/2-\sin\theta_{1})}{\cos\theta_{1}(\cos^{2}\theta_{1}+\beta^{2}\sin^{2}\theta_{1}+2\alpha_{2}\beta^{2})}\\ \frac{E_{1y}}{E_{S}}=\frac{\beta^{3}\cos\theta_{1}}{\left(\alpha_{2}+\alpha_{1}/2-\sin\theta_{1})\cdot (\sin^{2}\theta_{1}+\beta^{2}\cos^{2}\theta_{1}\right)} \end{array}\right.$$

The integration and incorporation of Equations (13) and (28) into Equation (27), and the subsequent connection with Equation (26), results in the following derived expression:

(28) $$\frac{E_{x}}{E_{s}}=\frac{\beta^{3}\xi_{00}L_{xd}}{(1+\xi_{00}\lambda_{1x})\cos\theta_{1}}$$

where *ζ*_02_ = *α*_3_^3^sin^2^*θ*_2_ + 3*α*_3_*α*_4_^2^cos^2^*θ*_3_ + *α*_4_^3^cos^2^*θ*_3_ + 3*α*_3_^2^*α*_4_sin^2^*θ*_2_, *ζ*_01_ = *α*_4_sin*θ*_3_ − *α*_3_cos*θ*_2_, *ζ*_03 =_ *α*_3_ cos^2^*θ*_2_ + *α*_4_ sin^2^*θ*_3_, *ζ*_00_ = *ζ*_01_/[cos*θ*_1_ × (*ζ*_02_ + *β*^2^*ζ*_03_)].

The REH cells and the REO cells possess equivalent spring stiffnesses, denoted as *K*_1*y*_ and *K*_2*y*_, respectively, in the *y*-direction. The aforementioned shapes also exhibit an equivalent total stiffness referred to as K*_y_*.

(29) $$K_{1y}=\frac{F_{y}}{\delta_{1y}}=\frac{E_{1y}\gamma L(\alpha_{2}+\alpha_{1}/2-\sin\theta_{1})}{\cos\theta_{1}},K_{2y}=E_{2y}\gamma L,K_{y}=\frac{E_{y}\gamma LL_{xd}}{\cos\theta_{1}}$$

The expression for *E_y_*/*E_s_* can be obtained by combining Equations (22) and (28), incorporating them into Equation (29), and subsequently connecting with Equation (26):

(30) $$\frac{E_{y}}{E_{s}}=\frac{(K_{1y}+K_{2y}).\cos\theta_{1}}{\gamma L.L_{xd}}$$

The deformation in the *y*-direction of a single cell during tensile deformation is characterized by *δ*_1*y*_ and *δ*_2*y*_.

(31) $$\delta_{1y}=2\varepsilon_{1y}Lcos\theta_{1},\delta_{2y}=2\varepsilon_{2y}Lcos\theta_{1}$$

Throughout the deformation process, the *y*-direction remains flat, and the strain energy *ε_y_* of the REHH cell in the *y*-direction is determined as follows:

(32) $$\varepsilon_{y}=\varepsilon_{1y}=\varepsilon_{2y}$$

The strain in the *x*-direction is derived as follows:

(33) $$\varepsilon_{y-x}=\frac{\delta_{1y-x}+\delta_{2y-x}}{L_{x}}$$

According to the formula of Poisson’s ratio, the Poisson’s ratio *v_yx_* of the REHH structures in the *y*-direction can be obtained by combining Equations (25) and (33) as follows:

(34) $$\nu_{yx}=-\frac{\varepsilon_{y-x}}{\varepsilon_{y}}=\frac{-u_{1y}\cos\theta_{1}+v_{2yx}(\alpha_{4}\sin\theta_{3}-\alpha_{3}\cos\theta_{2})}{L_{xd}}$$

where *u*_1*y*_ = 0.5 × (1 − *β*^2^) × sin^2^*θ*_1_/(sin^2^*θ*_1_ + *β*^2^cos^2^*θ*_1_).

## 3. Finite Element Analysis (FEA) and Experiment

### 3.1. Finite Element Analysis Section

The ANSYS workbench 2022 R2 software is utilized to perform static simulation analysis on the REHH structures composed of aluminium alloy. The material properties are defined as follows: *E_s_* = 33.3Gpa and *v_s_* = 0.33. A simulation model with parameters *L* = 10 mm, *α*_1_ = 1.8, *α*_2_ = 0.9, *β* = 0.05, *γ* = 0.5, *θ*_2_ = 50°, and *θ*_3_ = 60° was established using a 8 × 8 REHH element model with a grid size of 1 mm using beam 188, resulting in a total of 726,481 nodes and 93,575 elements. The specified boundary conditions were established as illustrated in Figure 6a below. The left boundary confined the displacement exclusively in the *x*-direction, while the lower boundary restricted it solely in the *y*-direction. On other hand, the right boundary remained unrestricted, while the upper boundary imposed a displacement of Δ*_uy_*= 10 mm. The displacement magnitude mentioned above was utilized to evaluate the elastic modulus *E_y_*/*E_s_* and the Poisson’s ratio *v_yx_* in relation to the *y*-direction of the REHH element. The FE model of a 8 × 1 REHH structure is depicted in Figure 6b. The model features a cell size of 0.1 mm, consisting of 104,574 nodes and 13,460 elements. The left and lower boundary conditions remain unchanged, while the upper boundary is unconstrained except for a prescribed displacement of Δ*_ux_*= 10 mm. This displacement is used to evaluate the elastic modulus *E_x_*/*E_s_* of the REHH element. By determining the total reactive force at the boundary nodes, it becomes possible to calculate both the in-plane elastic modulus and Poisson’s ratio of the REHH structures.

(35) $$\left\{\begin{array}{l} \varepsilon_{i}=\frac{\Delta u_{i}}{L_{i}}\\ \delta_{i}=\frac{F_{i}}{A_{j}} \end{array}\right.$$

The Young’s modulus *E_x_*/*E_s_*, *E_y_*/*E_s_*, and the Poisson’s ratio *v_yx_* can be determined through the following calculations:

(36) $$\left\{\begin{array}{l} E_{i}=\frac{\sigma_{i}}{\varepsilon_{i}}\\ \nu_{yx}=-\frac{\Delta u_{x}L_{y}}{\Delta u_{y}L_{x}} \end{array}\right.$$

where Δ*_u__x_* and Δ*_u__y_* represent the applied displacement, *F_i_* represents the total reaction forces exerted on the displaced boundary nodes, and *L_i_* and *A_j_*, respectively, denote the lengths and cross-sectional areas of the REHH cell in the *i* and *j* directions, corresponding to the *x* and *y* directions.

**Figure 7:**
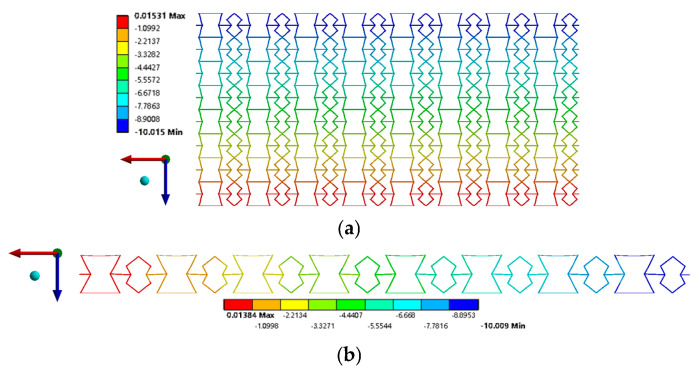
Displacement analysis results under tensile loading. (**a**) *y*-direction loading displacement analysis results, (**b**) *x*-direction loading displacement analysis results.

The present study presents the results of numerical simulations relevant to the tensile loading regime, as illustrated in Figure 7. The displacements of the FE model, both in the upper and constrained bounds, exhibit uniformity, as shown in Figure 7a, with remarkable homogeneity observed for displacements of Δ*_uy_*= Δ*_ux_*= 10 mm, respectively. Subsequently, the REHH structures are delineated using *θ*_1_ at varying angular positions, specifically at 10°, 20°, 30°, 40°, 50°, and 60°. The subsequent step involves the computation of the corresponding equivalent elastic moduli and Poisson’s ratio, followed by presenting simulation results in Table 1.

The following findings were established through a meticulous comparison between the simulated and theoretical results. (1) The relative errors (REs) between the simulated and theoretical results of the relative elastic moduli *E_x_*/*E*, *E_y_*/*E_s_*, and the Poisson’s ratio were no more than 7.03%, 9.5%, and 8.78%, respectively. (2) Within the angular range of 10° ≤ *θ*_1_ ≤ 30°, the simulated *E_y_*/*E_s_* ratio was lower than its theoretical counterpart, indicating a significant discrepancy. This can be attributed to a more pronounced concave configuration in the inner REO structures compared to the inner REH structures within this specified region, where the influence of the REO structures is predominant. Conversely, for 40° ≤ *θ*_1_ ≤ 360°, the increased concave angle of the cell walls on both sides of the REH structures exerts a more significant impact on the REH structures. (3) The errors occurring during this phase are characterized by extreme angles of the hybrid cytosol. In these phases, shape becomes more compact, resulting in non-uniform tensile deformation compared to the intermediate states. Consequently, even the tensile force obtained from the simulation is affected.

### 3.2. Experiments

**Figure 8:**
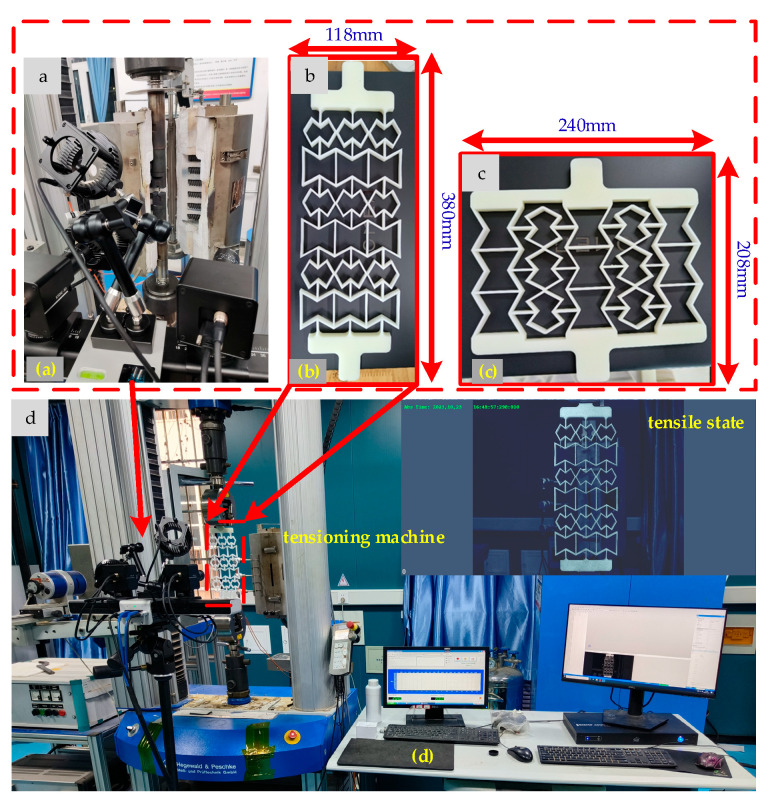
Tensile test rig. (**a**) High-speed camera, (**b**) y-axis stretch, (**c**) x-axis stretch, (**d**) test system.

Two samples of the REHH structures were manufactured using 3D printing technology, with the R4600 serving as the substrate. The elastic modulus *E_s_* is measured at 2600 Mpa. Both components exhibit a 3 × 3 hybrid honeycomb structure. The stretching model is illustrated in Figure 8.

The geometrical parameters, *α*_1_, *α*_2_, *α*_4_, *β*, *γ*, *θ*_1_, and *θ*_2_, were set at 0.9, 1, 0.8, 0.1, 0.5, 30°, and 50°, respectively. The uniaxial tensile experiments were conducted on a tensile machine equipped with a force transducer of capacity up to 100 kN at a constant tensile speed of 1 mm/min (Figure 8d). A high-speed camera was positioned vertically to capture the sample’s behavior during testing (Figure 8a). The *x*-direction sample fractures at a tensile length of 40.78 mm, whereas the *y*-direction sample failed at 11.19 mm. The double-concave cell wall structure significantly enhances the tensile performance of the *x*-direction sample compared to that of the *y*-direction one. The displacement and tension data were automatically recorded by the testing system throughout the stretching process, enabling the determination of equivalent elastic moduli and Poisson’s ratio using Equations (35) and (36), respectively. The mechanical properties were analyzed on three levels; theoretical, simulated, and experimental analyses were performed on 3 × 3 samples to obtain elastic modulus and Poisson’s ratio (Table 2). Comparative results between experimentally measured tensile force–displacement data and theoretical predictions show that the REs are no more than 0.63% (*E_x_*/*E_s_*), 7.4% (*E_y_*/*E_s_*), and 8.75% (*v_yx_*).

## 4. Discussion

**Figure 9:**
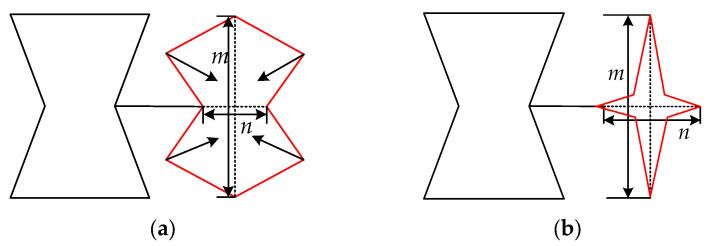
Cell structure transformation. (**a**) REO cell, (**b**) star-shaped cell.

The dimensionless quantities *m* and *n* are utilized to investigate the influence of variations in the angle of the concave octagon on Poisson’s ratio and elastic modulus. In cases where these two values differ, the concave octagon undergoes a transformation into a quadrangular star, resulting in a transition of Poisson’s ratio from negative to zero. By keeping *m* and *n* and decreasing the inner angle *θ*_2_ of the REO structures in such a way that its vertices become concave, it is possible to achieve parametric unification between the REHH structures and the FSHH structure, as depicted in Figure 9.

The dimensions in the *x* and *y* directions are expressed as follows:

(37) $$n=2(l\sin\theta_{3}-b\cos\theta_{2}),m=2(l\cos\theta_{3}+b\sin\theta_{2})$$

The proposed cell structure is subject to parametric simplification in order to fulfill the following relations:

(38) $$\alpha_{2}=\alpha_{1}/2=c/L=B/(2L)$$

### 4.1. Effect of Parameter θ_2_ on Cell Structure

**Figure 10:**
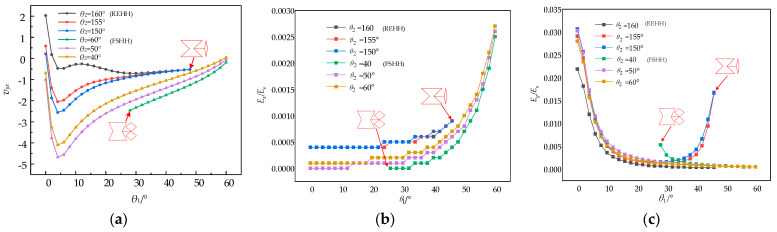
Poisson’s ratio and elastic modulus vs. *θ*_1_ for various internal angles *θ*_2_. (**a**) *v_yx_* vs. *θ*_1_, (**b**) *E_x_*/*E*_s_ vs. *θ*_1_, (**c**) *E_y_*/*E*_s_ vs. *θ*_1_.

The distinct ranges of *θ*_2_ and *θ*_3_ correspond to the geometries of the inner REO structures and the FSHH structures. The formation of an internal REH structures occurs when *θ*_2_ is smaller than 90°. The internal REO structures undergo a transformation into a four-point star, when *θ*_2_ is larger than 130° and smaller than 180°. For the REO structures, the values of *α*_1_, *α*_2_, *α*_4_, and *θ*_3_ are determined to be 1.8, 0.9, 0.7, and 50°, respectively, while for the four-point star, *θ*_3_ is set at 10°, with *β* = 0.075 and *γ* = 0.5 remaining constant. As *θ*_1_ varies under different conditions, there comes a critical point where any further change in the curve becomes insignificant. Figure 10 illustrates the impact of varying intracell angles *θ*_2_ and *θ*_3_ on the relative elastic moduli *E_x_*/*E_s_*, *E_y_*/*E_s_*, and Poisson’s ratio *v_yx_* based on Equations (28), (30) and (34).

The Poisson’s ratio *v_yx_* of the REHH structures in Figure 10a exhibits a significant decrease as *θ*_1_ increases, reaching its minimum at *θ*_1_ = 4°, followed by a gradual increase to approximately 0. The Poisson’s ratio of the REHH structures is consistent with that of the quadrangle. Moreover, the *v_yx_* value of the REHH structures is consistently lower compared to that of the quadrangle. Additionally, this value diminishes with an increase in *θ*_2_ due to the superior NPR characteristic of the REO structures over that of the quadrilateral star. The NPR characteristic of the hybrid unit cell composed of the REH structures is more pronounced compared to that of the REHH structures composed of quadrilateral stars.

The *x*-direction elastic modulus *E_x_*/*E_s_* of both cell combinations increases as *θ*_1_ increases, as shown in Figure 10b. Moreover, for the REHH structures, *E_x_*/*E_s_* is typically lower than that of the FSHH structures. The REHH structures exhibit a noticeable decline as the intracellular angule *θ*_2_, while *E_x_*/*E_s_* of the FSHH structures remains relatively unchanged. This can be attributed to the fact that the shapes of the REO structures undergo a more significant change in its shape with *θ*_2_ compared to the FSHH structures. The increase in *θ*_2_ leads to a corresponding growth in the equivalent length of the REH cells in the *x*-direction, thereby enhancing overall *x*-direction length of the REHH cells.

The *y*-direction elastic modulus *E_y_*/*E_s_* of the REHH structures decreases with the increase in *θ*_1_, as can be seen from Figure 10c. The *y*-direction elastic modulus *E_y_*/*E_s_* of the FSHH structures initially decreases and subsequently increases with the increase in *θ*_1_. The *E_y_*/*E_s_* of the REHH structures is typically positioned above the tetragonal star-shaped cell. The increase in *θ*_2_ leads to a reduction in the equivalent length of the REH cells in the *y*-direction. When *θ*_1_ increases to 40° or more, the FSHH structures gradually transforms into a rhombic shape due to reduced stretchability compared to its larger elastic modulus in the *y*-direction.

### 4.2. Effect of Parameters α_1_ and α_2_ on Cell Structure

**Figure 11:**
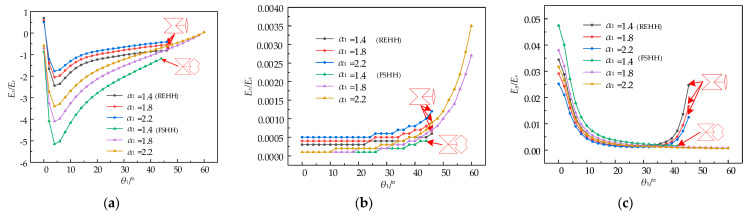
Poisson’s ratio and elastic modulus vs. *θ*_1_ for various wall aspect ratios *α*_1_ and *α*_2_. (**a**) *v_yx_* vs. *θ*_1_, (**b**) *E_x_*/*E_s_* vs. *θ*_1_, (**c**) *E_y_*/*E_s_* vs. *θ*_1_.

The comparative study of REHH structures and the FSHH structures revealed that, apart from variations in parameters *θ*_2_ and *θ*_3_, which directly influence the shapes of different cell elements, other parameters remained constant. When examining the impact of parameters *B* and *c*, it is advised to maintain *α*_4_ = 0.7, *β* = 0.075, *γ* = 0.5, as well as *θ*_2_ = 60° and *θ*_3_ = 50° in the context of an interior octagon. In the case of the FSHH structures, *θ*_2_ is equal to 155° and *θ*_3_ is equal to 10°. Figure 11 illustrates the effects of aspect ratios *α*_1_ and *α*_2_ on the relative elastic moduli *E_x_*/*E_s_*, *E_y_*/*E_s_*, and Poisson’s ratio *v_yx_* based on Equations (28), (30) and (34).

The Poisson’s ratios *v_yx_* of both REH structures and the REO structures increase with the increase in *α*_1_, as depicted in Figure 11a. However, it is observed that the rate of the curves decreases rapidly with the increase in *θ*_1_ and then gradually rises towards a position where the Poisson’s ratio is 0. The Poisson’s ratio *v_yx_* also increases with the increase in aspect ratio *α*_1_, while its magnitude decreases as *θ*_1_ increases. The relative equivalent elastic moduli, *E_x_*/*E_s_*, in the *x*-direction of both the REHH structures and the FSHH structures exhibit an increase with *θ*_1_, as observed in Figure 11b. *E_x_*/*E_s_* of the REHH structures is slightly smaller compared to the FSHH structures, and both structures show an increase in *Ex*/*Es* with *α*_1_. The relative elastic modulus *E_y_*/*E_s_* in the *y*-direction of the REHH structures decreases with an increasing *θ*_1_, as observed in Figure 11c. The relative elastic modulus *E_y_*/*E_s_* of the *y*-direction for the FSHH structures initially decreases with increasing *θ*_1_, but then exhibits a rapid increase around 40°, as explained in Figure 10. For the same value of *α*_1_ at a given *θ*_1_, *E_y_*/*E_s_* becomes smaller. Additionally, the REHH structures exhibit higher *E_y_/E_s_*.

### 4.3. Effect of Parameter on Cell Structure

**Figure 12:**
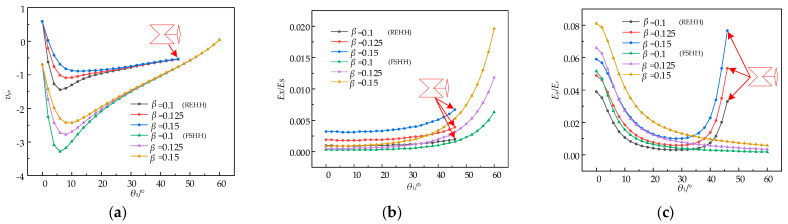
Poisson’s ratio and elastic modulus vs. *θ*_1_ for various wall aspect ratios β. (**a**) *v_yx_* vs. *θ*_1_, (**b**) *E_x_*/*E_s_* vs. *θ*_1_, (**c**) *E_y_*/*E_s_* vs. *θ*_1_.

The influence of wall thickness *t* is examined while maintaining *α*_1_ = 1.8, *α*_2_ = 0.9, *α*_4_ = 0.7, with the values of *θ*_2_ and *θ*_3_ being consistent with those utilized in the analysis of the aspect ratio’s impact on the REHH structures and the FSHH structures. The value of *β* is successively adjusted to 0.1, 0.125, and 0.15, respectively, for investigation purposes. Figure 12 illustrates the influence of the wall thickness ratio *β* on the relative equivalent elastic moduli, *E_x_*/*E_s_* and *E_y_*/*E_s_*, as well as the Poisson’s ratio, *v_yx_*.

The effect of the wall thickness ratio *β* on the Poisson’s ratio *v_yx_* is illustrated in Figure 12a; the *v_yx_* values of the REHH structures are lower than those of the FSHH structures. Both the REHH structures and the FSHH structures exhibit an increase in the Poisson’s ratio as the wall thickness ratio *β* increases, displaying a consistent trend of rapid decrease from 0° to 6°. The incorporation of the REHH structures results in a gradual convergence towards the Poisson’s ratio of zero. However, it is not possible for the FSHH structures to achieve the Poisson’s ratio of zero as it approaches its limiting shape.

The curves of the relative elastic modulus *E_x_*/*E_s_*, as depicted in Figure 12b, exhibit a relatively smooth profile. The increase in the wall thickness ratio *β* leads to a significant increase in *E_x_*/*E_s_*, resulting in a comparatively lower relative elastic modulus in the *x*-direction when compared to the FSHH structure. The relative elastic modulus *E_y_*/*E_s_* in the *y*-direction gradually decreases with increasing *θ*_1_ for the REHH structures, as depicted in Figure 12c. For the FSHH structure, there is initially a decrease from 0° to 34° followed by a rapid increase from 34° to 46° due to its transformation into a rhombic shape, which exerts the main influence. The greater the wall thickness ratio *β*, the higher the *E_y_*/*E_s_* value becomes for the REHH structures. Moreover, the REHH structures exhibit higher *E_y_*/*E_s_* values compared to the FSHH structures.

## 5. Conclusions

The present study proposed the REHH structures that demonstrates remarkable NPR properties by incorporating both internal REO structures and the REH structures. The theoretical model was developed to analyze the influences of three geometrical parameters of the cell element, namely the internal angle *θ*_2_, the aspect ratio *α*_2_, and the wall thickness ratio *β*. The analysis primarily focused on the surface mechanical properties. The validity of the theoretical derivation was confirmed through simulation and experimental results, leading to the following conclusions.

(1) The theoretical models of the elastic modulus *E_x_*/*E_s_*, *E_y_*/*E_s_*, and Poisson’s ratio *v_yx_* of the REHH structures were developed based on the Euler–Bernoulli beam model and Castigliano’s second theorem. The relative errors in the relative elastic modulus *E_x_*/*E_s_* and *E_y_*/*E_s_*, as well as Poisson’s ratio *v_yx_* between the simulation and theoretical results were no more than 7.03%, 9.5%, and −8.78%.
(2) The two samples of the REHH structures were fabricated using 3D printing technology, and an experimental setup was established for conducting a tensile test. The relative errors between the experimental and theoretical results of the relative elastic modulus *E_x_*/*E_s_* and *E_y_*/*E_s_*, as well as Poisson’s ratio *v_yx_* were no more than 0.63%, 7.4%, and 8.75%, thereby validating the accuracy of the theoretical and simulated models.
(3) Parametric studies reveal that the mechanical properties of the REHH structures exhibit a similar trend to the former FSHH structures as with the vertex angle *θ*_1_ of the concave hexagon increasing. The size ratio *E_x_*/*E_s_* of the REHH structures is typically smaller, while *E_y_*/*E_s_* is generally larger, with no significant disparity between them. However, in terms of NPR properties, the REHH structures demonstrate remarkable superiority compared to the FSHH structures. The parameters of the cell elements can be adjusted to achieve tuneable tensile properties.

## Figures and Tables

**Table 1:** Relative errors (REs) between theoretical results and simulated values.

	*Ex/Es*			*Ey/Es*			*v_yx_*		
*θ*_1_	Theoretical Results	Simulated Results	*REs*/%	Theoretical Results	Simulated Results	*REs*/%	Theoretical Results	Simulated Results	*REs*/%
10°	0.0000357	0.0000384	7.03	0.0023	0.00216	−6.48	−3.91	−4.2	−7.43
20°	0.000045	0.0000485	6.44	0.0008	0.000748	−6.95	−2.18	−2.39	−8.78
30°	0.0000691	0.000072	4.02	0.0005	0.000476	−5.04	−1.44	−1.54	−6.94
40°	0.000128	0.000134	4.68	0.0004	0.000425	5.88	−0.99	−1.07	−7.47
50°	0.000292	0.000305	4.2	0.0003	0.000328	8.53	−0.57	−0.62	−8.06
60°	0.000824	0.000883	6.68	0.0002	0.000221	9.5	0.0458	0.043	−6.51

**Table 2:** Comparison of simulated and experimental results.

	Simulated Results	Experimental Results	*REs*/%
*E_x_/E_s_*	0.0005366	0.0005403	0.63
*E_y_/E_s_*	0.00261	0.00248	7.4
*v_yx_*	−1.416	−1.54	8.75

## Data Availability

All data related to this study are available in the article.
